# Optogenetic induction of caspase-8 mediated apoptosis by employing *Arabidopsis* cryptochrome 2

**DOI:** 10.1038/s41598-023-50561-y

**Published:** 2023-12-27

**Authors:** Weiliang Mo, Shengzhong Su, Ruige Shang, Liang Yang, Xuelai Zhao, Chengfeng Wu, Zhenming Yang, He Zhang, Liuming Wu, Yibo Liu, Yun He, Ruipeng Zhang, Zecheng Zuo

**Affiliations:** Jlin Province Engineering Laboratory of Plant Genetic Improvement, College of Plant Science, Jilin University, Changchun, 130062 China

**Keywords:** Biological techniques, Biotechnology, Cell biology

## Abstract

Apoptosis, a programmed cell death mechanism, is a regulatory process controlling cell proliferation as cells undergo demise. Caspase-8 serves as a pivotal apoptosis-inducing factor that initiates the death receptor-mediated apoptosis pathway. In this investigation, we have devised an optogenetic method to swiftly modulate caspase-8 activation in response to blue light. The cornerstone of our optogenetic tool relies on the PHR domain of *Arabidopsis thaliana* cryptochrome 2, which self-oligomerizes upon exposure to blue light. In this study, we have developed two optogenetic approaches for rapidly controlling caspase-8 activation in response to blue light in cellular systems. The first strategy, denoted as Opto-Casp8-V1, entails the fusion expression of the* Arabidopsis *blue light receptor CRY2 N-terminal PHR domain with caspase-8. The second strategy, referred to as Opto-Casp8-V2, involves the independent fusion expression of caspase-8 with the PHR domain and the CRY2 blue light-interacting protein CIB1 N-terminal CIB1N. Upon induction with blue light, PHR undergoes aggregation, leading to caspase-8 aggregation. Additionally, the blue light-dependent interaction between PHR and CIB1N also results in caspase-8 aggregation. We have validated these strategies in both HEK293T and HeLa cells. The findings reveal that both strategies are capable of inducing apoptosis, with Opto-Casp8-V2 demonstrating significantly superior efficiency compared to Opto-Casp8-V1.

## Introduction

Apoptosis, a programmed cell death, is a complex process regulated by multiple genes that maintains organism development and internal environment homeostasis^1,2^. It is a cellular response to specific information from the environment such as information transmission, gene expression, and protein synthesis. Physiological and pathological stimuli can trigger apoptosis via three main pathways: death receptor, mitochondrial signals, and endoplasmic reticulum signals^3^. The death receptor pathway is mainly activated by caspase, a cysteine-containing aspartate-specific protease, which is the core component of this pathway^4^. In terms of mechanism, cell apoptosis is controlled through two pathways: the extrinsic/death receptor pathway and the intrinsic/mitochondrial pathway. Both of these pathways involve apoptotic initiators and caspases, which are members of the cysteine-aspartic protease family capable of cleaving substrate proteins that promote cell death. Specifically, the extrinsic/death receptor pathway includes Caspase-8, while the intrinsic/mitochondrial pathway involves Caspase-9 and Caspase-3/-7^5^. Caspase 8, a key promoter in the death receptor-mediated apoptosis pathway, is activated through oligomerization and self-cleavage, which triggers downstream caspases and ultimately leads to cell apoptosis^6^. Therefore, the controlled oligomerization of caspase 8 can achieve automatic regulation of apoptosis. However, to regulate caspase 8 cellular function precisely, the intensity, location, and duration of signaling events need to be modulated, especially for the activation process, in which the oligomerization of caspase 8 needs to be under microscale control for downstream ultrasensitive digital signaling responses.

It has long been established that caspases can cleave and activate themselves or other proteins through their aspartate-specific proteolytic enzyme activity. To induce aggregation activation of Caspase 8, a fusion was created by linking the Caspase 8 precursor with the dimerization domain FKBP (the binding domain of FK506). When the dimerization ligand FK1012 homodimerizes, the fused Caspase 8 precursor also polymerizes, leading to dimerization of the Caspase 8 precursor and subsequent activation^7,8^. Dixit and Salvesen consolidated these findings under the Induced Proximity Model (IPM)^9^. According to this model, when zymogens are recruited to form the Death-Inducing Signaling Complex (DISC), the local zymogen concentration increases, resulting in the activation of caspase 8 precursors. Research has demonstrated that these stable homodimers undergo self-activation through cleavage by their own proteases, while also being susceptible to the action of neighboring dimers. The activation of the Caspase 8 precursor requires two cleavages. The first cleavage occurs at residue D374, yielding the p43/p41 and p12 subunits. Subsequent cleavages at residues D216 and D384 give rise to the p26/p24, p18, and p10 subunits. The p18 subunit and the p10 subunit then form a heterotetramer and disengage from the DISC^10^. This heterotetramer represents active Caspase 8, which is eventually released into the cytoplasm to initiate apoptosis^11,12^.

Light-induced cell death induction offers several notable advantages compared to methods that using chemical compounds: faster and easier signal transmission, precise control of the strength and duration of cell death stimuli by altering light dosage and duration, and the ability to restrict the induction of cell death to selected cells or tissues of interest. Various methods using optogenetics exist to activate proteins. Recently, light-sensitive protein domains, such as LOV2 (Light-Oxygen-Voltage 2), have been used as tools to catalyze the separation of caspase-3 and -7 subunits when activated^13,14^. Certainly, there have been studies involving the fusion of caspase 8 with mutant variants of the N-terminal PHR of the blue light receptor CRY2 to induce cell apoptosis. These studies have provided alternative approaches for blue light-regulated apoptosis in HEK293T cells and zebrafish cells^15^. The exploration of the blue light-dependent interaction between CRY2 and CIB1 to regulate caspase8 activity in HeLa cells remains limited. Here, we suggested a method for modulating cell apoptosis through the CRY2-CIB1 interaction, aiming to diversify blue light-induced cell apoptosis approaches and may offer a fresh perspective on employing optogenetic tool in cancer treatment.

## Materials and methods

### Plasmids constructs

For constructing Opto-Caspase8 plasmids, a pCI (neo) (Promega, E1841) was used. Myc, Flag or GFP was inserted into pCI (neo) with EcoRI restriction site to construct pCI (neo) flag, pCI (neo) myc, and pCI (neo) GFP. The coding sequences of CRY2 PHR or CIB1 N-terminal domain were bridged with Caspase8 by overlapping PCR and then inserted into the pCI (neo) Flag, pCI (neo) Myc or pCI (neo) GFP plasmid using XbaI /XmaI restriction sites to obtain pCI (neo) Myc-PHR-Caspase8, pCI (neo) Flag-PHR-Caspase8 and pCI (neo) GFP-PHR-Caspase8. In order to exclude the effect of endotoxin for apoptosis so all of the plasmids were extracted using Qiagen (12943) plasmid extraction kit. Primers used for vector construction are listed in Supplementary Table 1.

### Human cell culture and transfected

HEK-293T cells (ATCC, ATCC®CRL-11268TM) or HeLa cells (ATCC, CRM-CCL-2™) were cultured in DMEM (invitrogen, 10569-044) supplemented with 10% (v/v) FBS (Invitrogen 10100147), 100 U/ml penicillin, and 100 mg/ml streptomycin (Hyclone, SV30010) in humidified 5% (v/v) CO2 in air, at 37 °C. Cells were seeded at a density of 3 × 10^5^ cells per well in a six-well plate and transfected using Lipofectamine 3000 transfection methods as manual instructions described.

### Co-immunoprecipitation (Co-IP) assays

Transfected cells were exposed to blue light (30 μmol m^−2^ s^−1^) or kept in the dark for the indicated time before being lysed. Cell pellets were lysed using Pierce IP Lysis Buffer (87787, Pierce) supplemented with 1× EDTA-free Protease Inhibitor Cocktail Tablets (4693159001, Roche), and then incubated on ice for 15 min. After centrifugation at 14,000×*g* for 10 min at 4 °C, the supernatant was mixed with 20 μl of GFP trap beads and incubated with vertical blending at 4 °C for 2 h. The beads were washed 5 times with washing buffer [20 mM HEPES (pH 7.5), 40 mM KCl, 1 mM EDTA] and denatured by thoroughly mixing with 30 μl of 4× Loading buffer and heating at 100 °C for 10 min. Co-IP samples were detected by Western blot and probed with anti-GFP (MBL, 598) or anti-Flag (MBL, M185-3S), respectively.

### Caspase8 cleavage assay

Transfected HeLa or HEK293T cells were either exposed to blue light (30 μmol m^−2^ s^−1^) or kept in the dark for the indicated time. Then, the cells were dissociated from the dishes with TrypLE™ Express (1x) (Gibco, 12605-028) at 37 °C for 5 min. After centrifugation at 800×*g* for 5 min, the supernatant was discarded. The cell pellets were lysed with Pierce IP Lysis Buffer (87787, Pierce) supplemented with 1× EDTA-free Protease Inhibitor Cocktail Tablets (4693159001, Roche) and incubated on ice for 15 min. The mixtures were centrifuged at 14,000×*g* for 10 min at 4 ℃ to remove cell debris. The supernatants were boiled with 4× Loading buffer for 10 min. Then, the samples were detected by western blot and probed with anti-Caspase8 (Abcam, ab32397), anti-Caspase3 (MBL, M097-3), and anti-Actin (MBL, M177-3). The Western blot band intensities were analyzed with ImageJ software, and all the band intensities were normalized to actin.

### Flow cytometry

Transfected HeLa cells were exposed to blue light (50 μmol m^−2^ s^−1^) for 3 h. The cells were then labeled with Alexa Fluor 488 annexin V and propidium iodide (PI) according to the protocol of the Alexa Fluor 488 Annexin V/Dead Cell Apoptosis Kit (V13241, Invitrogen) for flow cytometry. The labeled cells were analyzed using the BD Aria II system. The relative cell death rate was calculated as the number of apoptotic cells in blue light divided by the number of apoptotic cells in the dark. For FAM-LETD-FMK caspase-8 assay in Hela cells using flow cytometry, the transfected HeLa cells were exposed to blue light (30 μmol m^−2^ s^−1^) for 12 h, stained with the Image-iT LIVE Green Caspase-8 Detection Kit (I35105, Invitrogen) then the labeled cells were analyzed using the BD Aria II system.

### Microscope imaging

For the CRY2-PHR-mCherry oligomerization assay in HEK293T cells, the transfected cells were exposed to 50% laser power of the Zeiss confocal LSM880 for the indicated time. Image analysis was performed using Zen software (Zeiss) and processed with Adobe Photoshop. For active Caspase8 detection by microscope, the transfected HEK293T cells were exposed to blue light (30 μmol m^−2^ s^−1^) for 3 h or kept in the dark and stained with the Image-iT LIVE Green Caspase-8 Detection Kit (I35105, Invitrogen). For the DAPI-labeled detection assay, the transfected HeLa cells were exposed to pulse blue light (50 μmol m^−2^ s^−1^) for 12 h (10 min on/10 min off) or kept in the dark and stained with DAPI (10 μg/ml). The images were obtained using the Zeiss Observer A1 reverse fluorescence microscope. Image analysis was performed using Zen software and processed with Adobe Photoshop. Fluorescent intensity was analyzed and obtained using ImageJ software.

### Quantification and statistical analysis

All data were collected using Excel and analyzed using ANOVA with a two-tailed Student's t-test for statistical significance.

## Results

### Design and creation of opto-caspase8

**Figure 1:**
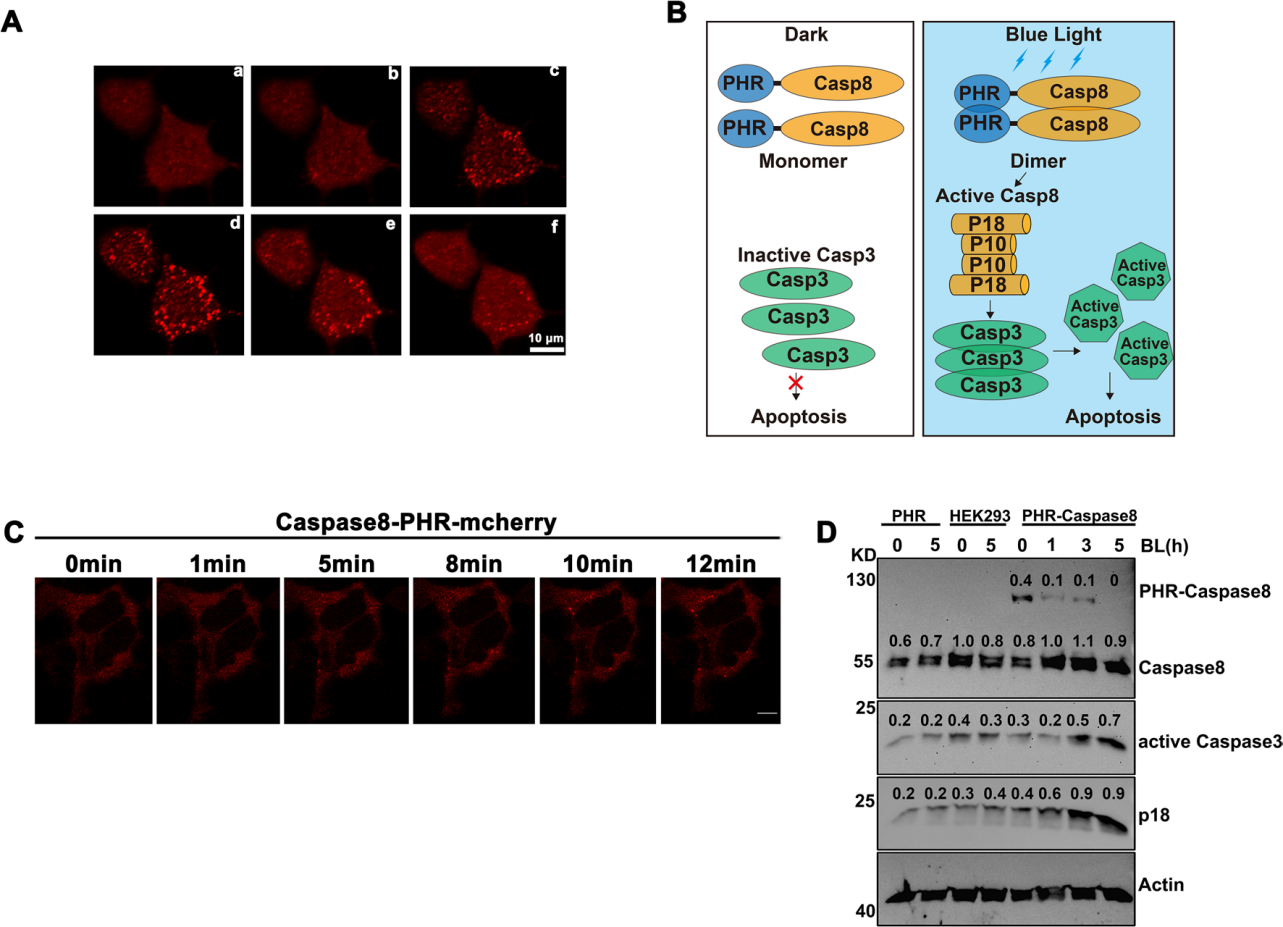
Design of optogenetic tools to control cell apoptosis by blue light. (**A**) Blue light induces CRY2-PHR-mcherry oligomerization in HEK293T cells. a. dark; b. blue light 1 min; c. blue light 5 min; d. blue light 10 min; e. blue to dark 5 min; e. blue to dark 10 min. (**B**) The photo-responsive region of *Arabidopsis thaliana* cryptochrome 2 (PHR, amino acid 1–498) is fused with Caspase8. In the dark, the engineered PHR-Casp8 exhibits monomer. Upon blue light illumination, PHR drives dimerization of PHR-Casp8 and promoters the apoptosis. Casp8, Caspase8; Casp3, Caspase3; PHR, the N-terminal domain of CRY2. (**C**) Clustering of caspase-8-PHR-mCherry in response to blue light in HEK293T cells, bar = 10 μm. (**D**) Blue light actives Opto-Caspase8-V1 in HEK293 T cells. Transfected HEK293T cells were crushed by Pierce IP lysis buffer, total cell lysates were analyzed by western blotting probed with anti-Caspase8, anti-Caspase3, actin was used as a loading control.

In previous studies, it has been found that CRY2 or its PHR domain (CRY2PHR) underwent oligomerization in a blue light dependent manner^16–18^. To confirm CRY2PHR can underwent oligomerization in human cells, we fused PHR to mcherry and transfected into HEK293T cells, and we found that PHR-mcherry can cluster in a blue light dependent manner (Fig. 1A) as expected. To control the caspase8 mediated signaling pathway with blue light, we first fused the PHR domain of CRY2 with caspase8 (PHR-Caspase8) as the new optogenetic tool (Opto-Casp8-V1). As illustrated in Fig. 1B, the PHR-Caspase8 was monomer and kept inactive in darkness. Under blue light irradiation, the PHR-Caspase8 dimerized and oligomerized because of the blue light specific oligomerization of PHR. The oligomerized PHR-Caspase8 then self-cleavaged and released the activated caspase8 domain (P18 and P10) to active downstream caspase3 and promote cell apoptosis. To further confirm the ability of the fusion protein to aggregate, we assessed the aggregation of caspase-PHR-mCherry in HEK293T cells following blue light induction. The results demonstrated that it can indeed aggregate (Fig. 1C). To confirm this process, we transferred the constructed Opto-Casp8-V1 (PHR-Caspase8) including the PHR-only and empty vector control into HEK293T cells, respectively, and treated them with blue light for 0, 1, 3 and 5 h respectively, lysed the cells, and detected the protein level by western. We analyzed the status of PHR-Caspase8, the activated P18 and the downstream protein from dark to blue light irradiation. As excepted, the abundance of the precursor PHR-Caspase8 was decreased after blue light irradiation (Fig. 1D), which suggested the PHR-Caspase8 could self-cleavage and consume in blue light driven by the oligomerization of PHR. On the other hand, the activated P18 of caspase8 and the activated caspase3 were accumulated from dark to blue light irradiation (Fig. 1D), suggesting that the activity of PHR-Caspase8 could be controlled by blue light to active the downstream signaling pathway in blue light.

### Blue light induce opto-caspase8 cluster and self-cleavage

**Figure 2:**
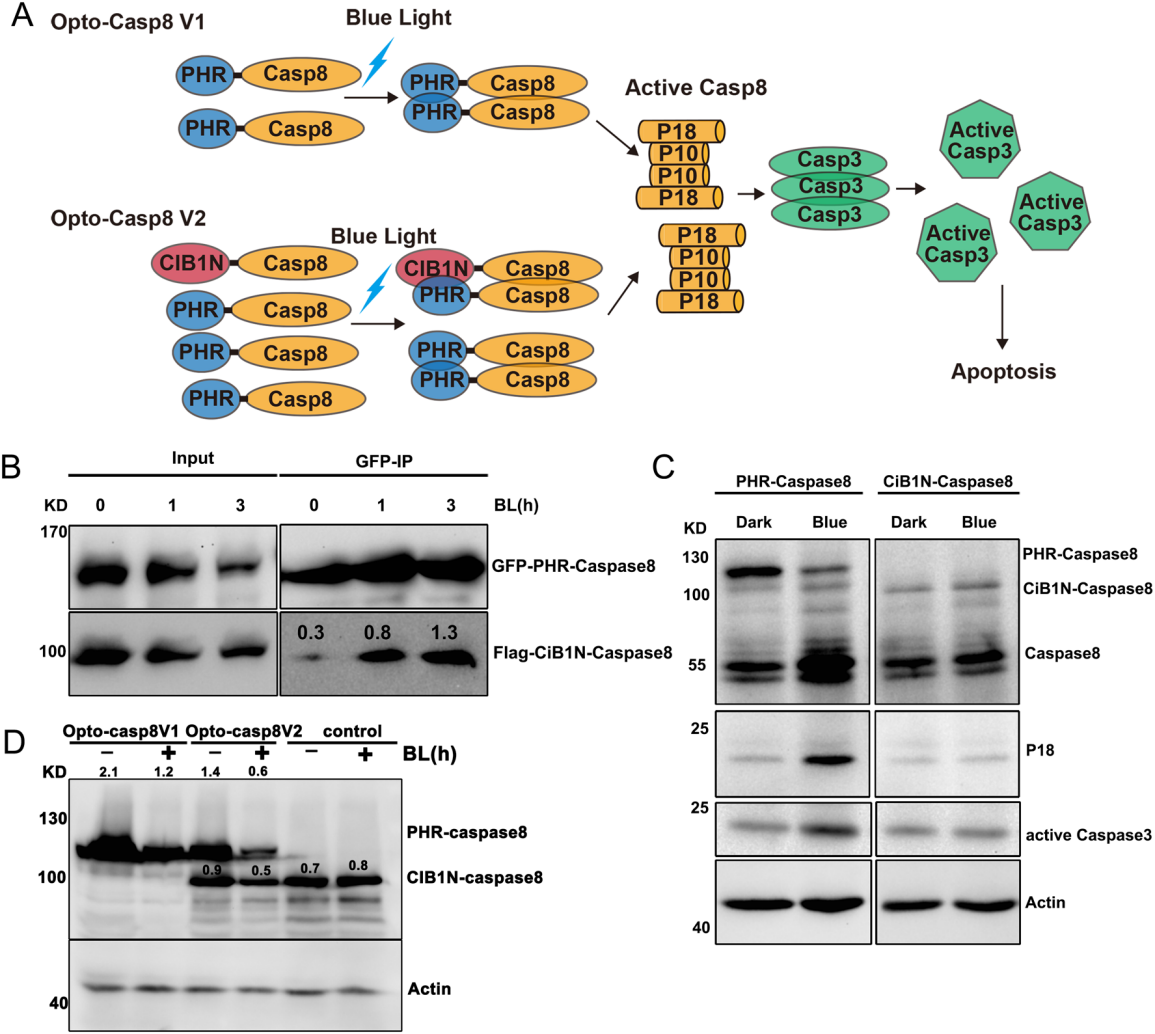
Blue light trigger Opto-Caspase8 cleavage and activation. (**A**) N-terminal part of cryptochrome interacting basic-helix–loop–helix protein CIB1 (amino acids 1–170, CIB1N) are fused with Caspase8, PHR-Caspase8 is marked as Opto-Casp8 V1 and PHR-Caspase8/CiB1N-Caspase8 as Opto-Casp8 V2. CIB1N, the N-terminal domain of CIB1. (**B**) Co-IP assay showed blue light enhanced the interaction of Opto-Caspase8 V2 in HEK293 T. Transfected HEK293T cells were treated with blue light (30 μmol m^−2^ s^−1^) for indicated time before lysed. The immunoprecipitation signals were probed by anti-GFP or anti-Flag, respectively. (**C**) Blue light actives Opto-Caspase8 V1 in HeLa cells. (**D**) Cleavage efficiency of caspase8 between Opto-Caspase8 V1 and Opto-Caspase8 V2 in Hela cells.

Building upon earlier findings that the *Arabidopsis *blue light receptor CRY2 can interact with the transcription factor CIB1 in a blue light-dependent manner, we created an optogenetic tool for caspase8 by fusing the N-terminal domain of CIB1 (amino acids 1–170) with caspase8 to generate CIB1N-caspase8, which was then combined with PHR-caspase8 to produce Opto-Casp8-V2 (Fig. 2A). This design ensured that the optogenetic system could effectively regulate the caspase8-mediated signaling pathway via blue light. To validate this approach, we transfected HEK293T cells with GFP-PHR-caspase8/Flag-CIB1N-caspase8 (Opto-Casp8-V2 cassette), treated the cells with blue light, and subsequently lysed them. We performed immunoprecipitation (IP) with GFP trap and observed that all the proteins of GFP-PHR-caspase8/Flag-CIB1N-caspase8 underwent blue light-dependent cleavage and interacted in a blue light-enhanced manner in co-IP (Fig. 2B). These results indicate that Opto-Casp8-V2 can effectively function in cells, similar to Opto-Casp8-V1.

We next evaluated the effectiveness of PHR-caspase8 and CIB1N-caspase8 in the human cervical cancer cell line HeLa. We firstly transfected HeLa cells with PHR-caspase8 and CIB1N-caspase8, treated them with blue light, and observed that PHR-caspase8 exhibited highly efficient self-cleavage and induced downstream accumulation of caspase3, whereas CIB1N-caspase8 could not be activated by blue light (Fig. 2C). We compared the cleavage efficiency mediated by Opto-Casp8-V1 and Opto-Casp8-V2 in HeLa cells and found that Opto-Casp8-V2 demonstrated significantly more effective self-cleavage and consumption than Opto-Casp8-V1 under blue light, indicating that CIB1N-caspase8 could enhance the activation of PHR-caspase8 in the Opto-Casp8-V2 optogenetics cassette (Fig. 2D). This finding further suggests that, in addition to the oligomerization of CRY2, the CRY2-CIB1 protein–protein interaction can further promote the activation of precursor caspase8 in blue light.

### Optogenetic control of caspase8-mediated apoptosis and programmed cell death

**Figure 3:**
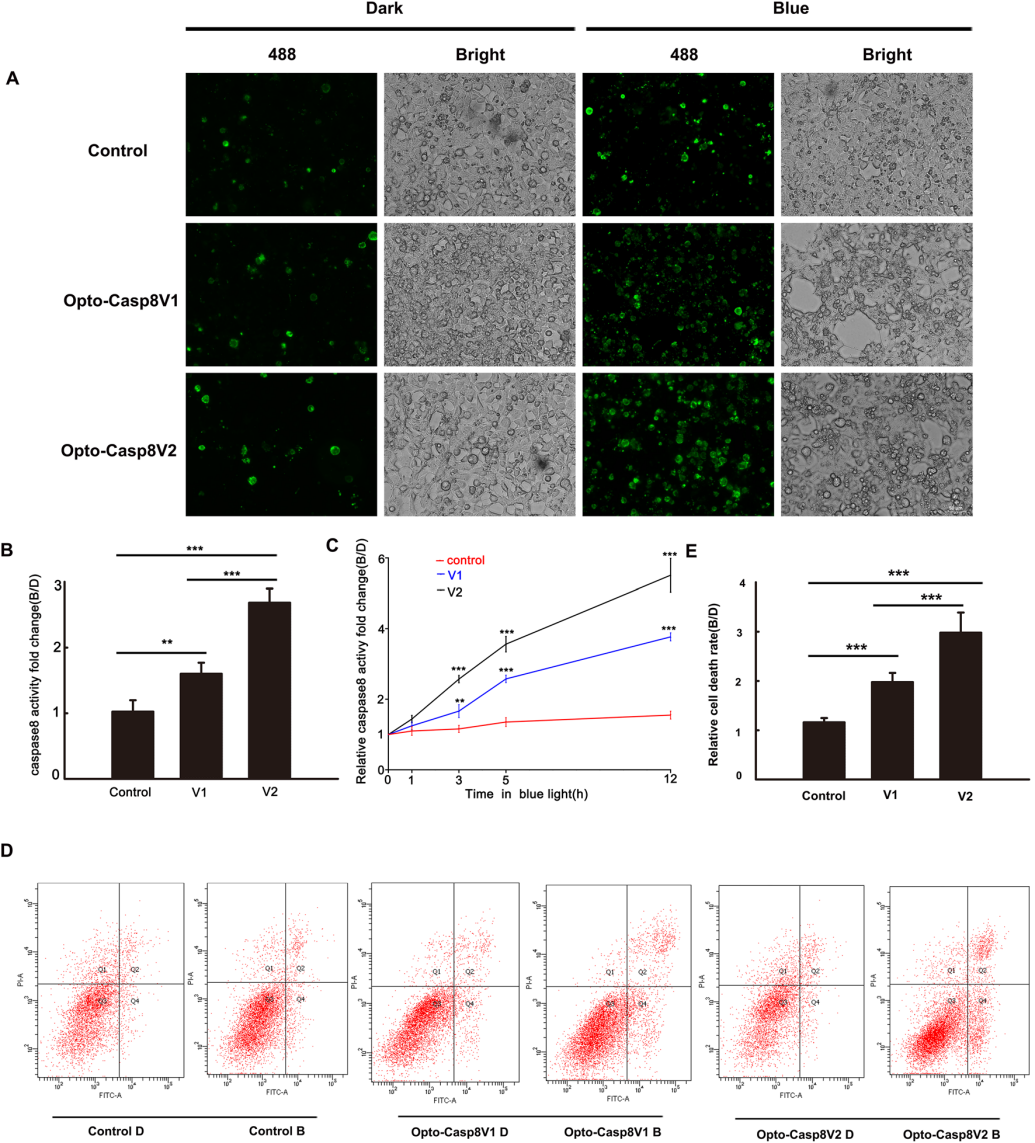
Blue light dependent apoptosis activated by Opto-Caspase8. (**A**) Active caspase8 was detected by fluorescence microscope labeled by FAM-LETD-FMK caspase-8 in HEK293T cells. Blue light (50 μmol m^−2^ s^−1^ for 3 h) treated transfected cells were incubated with FAM-LETD-FMK caspase-8 before capturing pictures, CiB1N-Caspase8 and the empty vector pci myc were co-transfected as control. (**B**) Caspase8 activity analysis in (**A**), activities = fluorescent intensity of caspase8 in blue/fluorescent intensity of caspase8 in dark. Data are presented as mean ± SD (n = 3). Student’s t test: ***p < 0.001. (**C**) Time course of FAM-LETD-FMK labeled caspase-8 activity assay in HEK293T cells. Same operation were conducted as showed in (**A,B**). Data are presented as mean ± SD (n = 3). Student’s t test: ***p < 0.001. (**D**) Cell viability analysis in HeLa cells expressed Opto-Caspase8 V1 or Opto-Caspase8 V2 by flow cytometry. Alexa Fluor® 488 annexin and PI was used to detect apoptosis cells. The transfected cells were either shielded or exposed to continuous blue light (50 μmol m^−2^ s^−1^) for 3 h. (**E**) Relative cell death rate analysis in (**D**). Rate = number of apoptosis cells in blue/number of apoptosis cells in dark. Data are presented as mean ± SD (n = 3). Student’s t test: ***p < 0.001.

We utilized FAM-LETD-FMK caspase-8 to evaluate the effectiveness of caspase-8 activation in live cells using our Caspase8 optogenetic tool. Plasmids encoding Opto-Caspase8-V1 and Opto-Casp8-V2 were transfected into HEK293T cells, and after blue light stimulation, the cells were labeled with FAM-LETD-FMK and imaged using an inverted microscope (Zeiss Axio Observer A1). Our results demonstrated that Opto-Casp8-V1 and Opto-Casp8-V2 induced greater activation of caspase-8 in live cells than cells transfected with the CIB1N-Caspase8 control (Fig. 3A,B). Moreover, Opto-Casp8-V2 exhibited significantly higher activation efficiency than Opto-Casp8-V1 (Fig. 3A,B), confirming the usefulness of CIB1N-Caspase8 as an optogenetic tool for caspase8 signaling pathway activation. We further examined the morphological changes in cells transfected with Opto-Casp8-V1 and Opto-Casp8-V2, and our data showed that Opto-Casp8 induced more active Caspase8 in HEK293T cells after effective self-cleavage under blue light, resulting in apoptosis (Fig. 3A,B). A time-course assay of FAM-LETD-FMK caspase-8 activity demonstrated a positive correlation between blue light treatment duration and FAM-LETD-FMK caspase-8 activity (Fig. 3C), further supporting the ability of Opto-Casp8 to trigger apoptosis in human cells.

**Figure 4:**
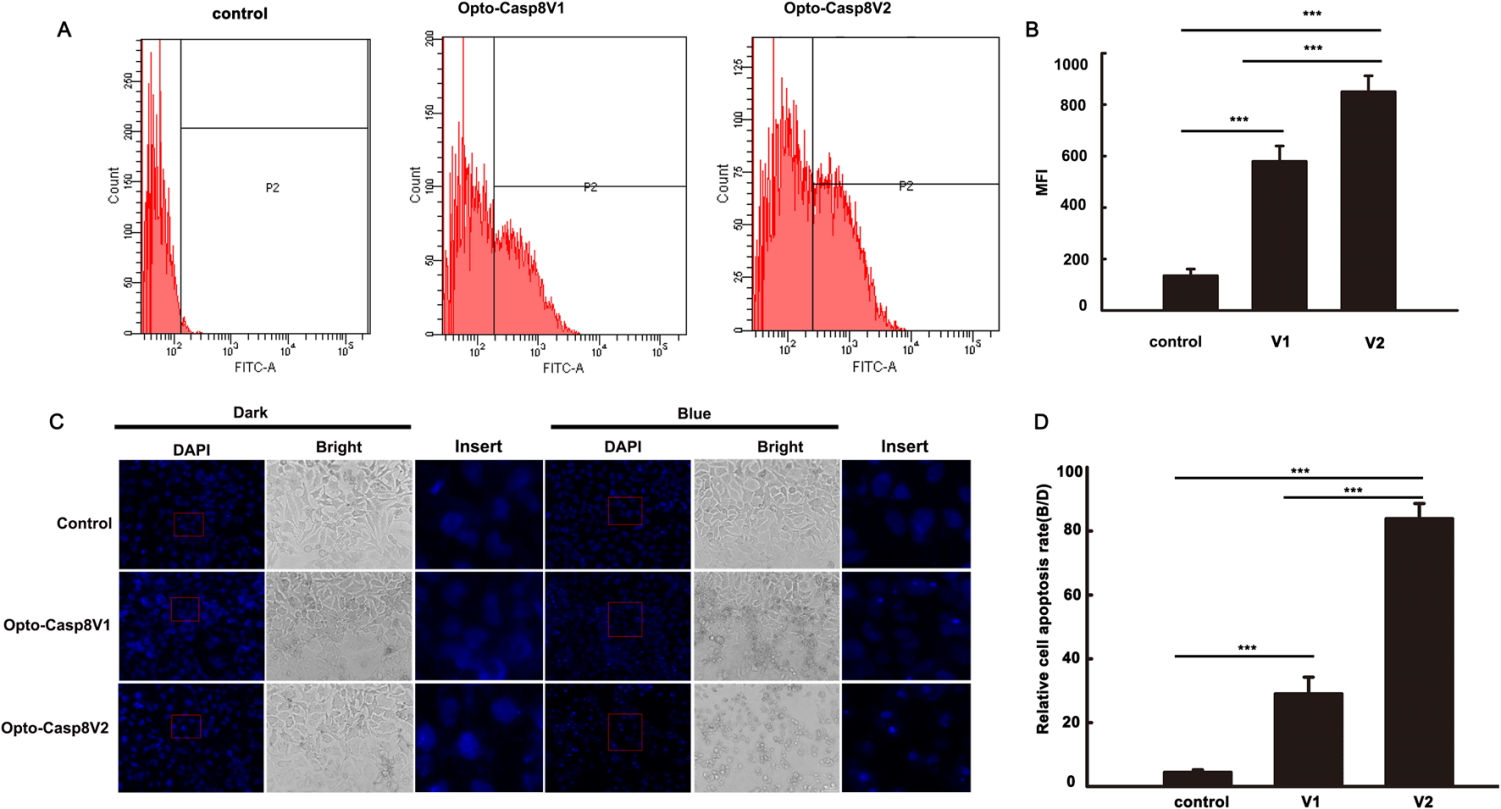
Opto-Caspase8 cause cell death in HeLa cells. (**A**) Detecting the caspase-8 activity with FAM-LETD-FMK with flow cytometry in HeLa cells under blue light, the empty vector was transfected as control. (**B**) Mean fluorescence intensity (NFI) of (**A**). Data are presented as mean ± SD (n = 3). Student’s t test: ***p < 0.001. (**C**) The transfected cells were either shielded or exposed to pulse blue light (50 μmol m^−2^ s^−1^) for 12 h and incubated with DAPI, after washing for 3 times, pictures were obtained by Zeiss Axio Observer A1 microscope, CiB1N-Caspase8 and the empty vector pci myc were co-transfected as control. (**D**) Relative cell apoptosis rate analysis of (**A**), rate = number of condensed nuclei in blue/number of condensed nuclei in dark. Data are presented as mean ± SD (n = 3). Student’s t test: ***p < 0.001.

To investigate the efficacy of Opto-Casp8-V1 and Opto-Casp8-V2 in inducing cell apoptosis via the caspase8-mediated signaling pathway under blue light, we employed flow cytometry to monitor apoptosis in cells induced by Opto-Casp8-V1 and Opto-Casp8-V2. During apoptosis, phosphatidylserine (PS) flips to the outer layer of the lipid membrane^19,20^. Annexin-V is a Ca^2+^-dependent phospholipid-binding protein with a molecular weight of 35–36 kD, which exhibits high affinity for phosphatidylserine^21^. The combination of phosphatidylserine and Annexin-V exposed on the lateral side of the cell is indicative of cell apoptosis^22^. Therefore, we used Alexa Fluor® 488 Annexin-V/PI with flow cytometry to verify cell apoptosis. Plasmids encoding Opto-Casp8-V1 and Opto-Casp8-V2 were transfected into HeLa cells, and after blue light stimulation, Alexa Fluor® 488 Annexin-V and PI-labeled cells were detected by flow cytometry. Our results indicated that compared to the control group (CIB1N-Caspase8), Opto-Casp8-V1 and Opto-Casp8-V2 significantly promoted cell apoptosis under blue light (Fig. 3D). Additionally, Opto-Casp8-V2 exhibited a stronger effect on promoting cell apoptosis than Opto-Casp8-V1 (Fig. 3D,E), which is consistent with the previous results of cleavage efficiency. Similarly, we employed flow cytometry to assess caspase-8 activity in HeLa cells following transfection with Opto-Casp8-V1 and Opto-Casp8-V2 plasmids. The results revealed that, after transfection with Opto-Casp8-V2, its activity was significantly higher than Opto-Casp8-V1, and both Opto-Casp8-V1 and Opto-Casp8-V2 activities were noticeably higher than the control (Fig. 4A,B). This indicates that Opto-Casp8-V1 and Opto-Casp8-V2 are capable of inducing cell apoptosis in HeLa cells.

The process of apoptosis involves multiple stages of morphological changes. In the initial stage, chromatin condenses and separates, distributing along the nuclear membrane. The cytoplasm also undergoes shrinkage, but its membrane remains intact with selective permeability. In the late stage of apoptosis, the chromatin breaks into fragments of varying sizes, which aggregate with organelles such as mitochondria and are surrounded by the inverted cell membrane. Subsequently, they gradually separate to form condensed nuclei^23,24^. In light of these observations, we employed DAPI nuclear staining to detect programmed cell death induced by Opto-Casp8. Our results demonstrated that Opto-Casp8 V1 and Opto-Casp8-V2 triggered the shrinkage of a large number of nuclei to form condensed nuclei, ultimately promoting cell death (Fig. 4C,D). In summary, our optogenetic tools (Opto-Casp8-V1 and Opto-Casp8-V2) exhibit caspase-8 activation activity in a blue light-dependent manner, subsequently activating downstream proteins to control cell apoptosis with blue light.

## Discussion

Caspase 8 is a critical factor in the apoptotic program of cell death, and its activation is essential for the functionality of the apoptotic pathway. Resistance to cell apoptosis is not only a hallmark of cancer but also significantly associated with enhanced drug resistance in tumor cells^25^. Targeted cell apoptosis is considered a highly promising approach in cancer treatment^26^. In this study, we developed Opto-Casp8 as a genetic tool for regulating cell apoptosis. Our results demonstrate that Opto-Casp8 induces apoptosis in HEK293T and HeLa cells, consistent with previous studies using Opto-BAX with CRY2-CIB1 or Opto-Caspase7 to regulate cell apoptosis^13,14^. However, our study specifically focuses on Caspase8, an upstream protein in the apoptosis pathway, rather than downstream proteins such as BAX (which regulates the mitochondrial pathway) or Caspase3. Caspase8 mediates cell apoptosis not only through the mitochondrial pathway but also through other caspases, such as Caspase3/7/9, providing a wider regulatory range.

Recent reports suggest that Caspase-8 not only acts as the initiator of extrinsic apoptosis but also as a molecular switch for necroptosis and pyroptosis^13^. Compared to optogenetics tools based on BAX or Caspase3, our optogenetics cassette (Opto-Casp8-V1 and Opto-Casp8-V2) has more widespread applications. Our optogenetics tool can precisely control Caspase-8 activity to induce cell apoptosis and utilize blue light to control the activation of the inflammasome and induction of pyroptosis when apoptosis and necroptosis are compromised. In the future, we expect that our optogenetics tools can regulate Caspase-8-mediated signaling pathways to tailor specific immune responses against pathogens and switch different modes of cell death. However, further research is needed to determine the most effective optogenetic tools and conditions and how to apply them to different types of cancer.

In addition, there is research exploring the use of a similar approach, fusing a mutant CRY2PHR oligo with caspase8, thereby regulating cell apoptosis through blue light^15^. What sets our study apart is that we not only validated the feasibility of PHR-caspase8 in HEK293T cells but also in cancer cells, specifically HeLa cells. The results indicated its ability to induce apoptosis in HeLa cells, broadening the potential applications of this tool and may providing an additional option for optogenetic cancer therapy.

Another crucial point of discussion is the safety of optogenetic methods. When using these tools, it is essential to ensure that they do not have adverse effects on healthy cells, and a thorough investigation of potential risks is required. Furthermore, the impact of different types of light sources and illumination conditions on cells needs to be taken into account to determine the optimal experimental conditions. Optogenetics also offers a novel approach to the spatiotemporal control of cell apoptosis research. By adjusting the timing and location of light exposure, researchers can precisely regulate the occurrence of cell apoptosis, contributing to a better understanding of the apoptotic processes in different cell types and tissues. In conclusion, optogenetics, as a novel approach to controlling cell apoptosis, presents exciting opportunities. However, further research is required to address the challenges and safety concerns in its application.

### Supplementary Information

Supplementary Information.

## Data Availability

The datasets used and/or analysed during the current study available from the corresponding author on reasonable request.
