# Supplementary material for: Optogenetic induction of caspase-8 mediated apoptosis by employing Arabidopsis cryptochrome 2
Source: Sci Rep. 2023 Dec 27;13:23067. doi: 10.1038/s41598-023-50561-y (PMC10754905; doi:10.1038/s41598-023-50561-y)
Supplement: Supplementary file 1 — Supplementary Information. [file 41598_2023_50561_MOESM1_ESM.pdf]

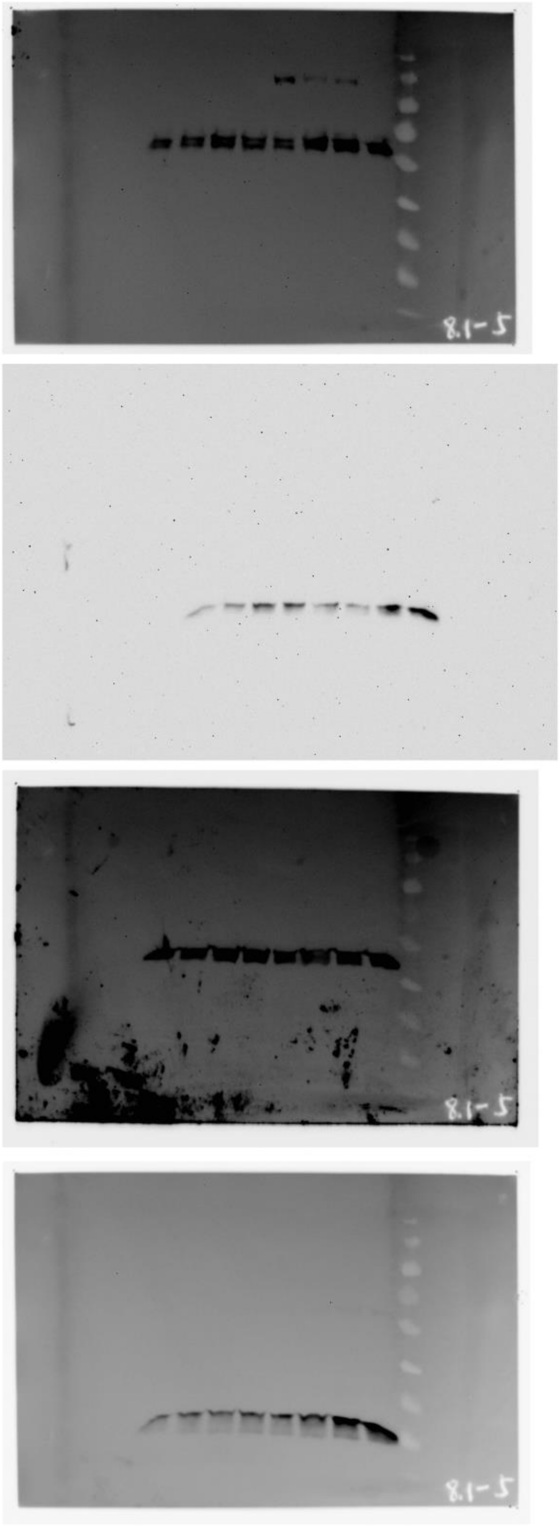

FIG1C

Short exposure

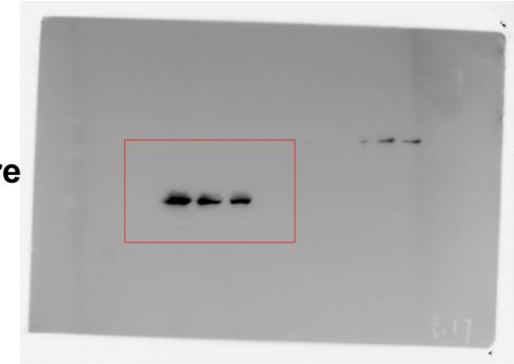

unstripped GFP-PHR-caspase8

Flag-cib1n-caspase8

Anti-Flag

Long exposure

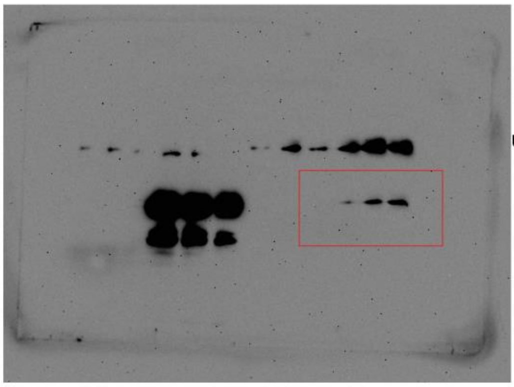

unstripped GFP-PHR-caspase8

Flag-cib1n-caspase8

Anti-Flag

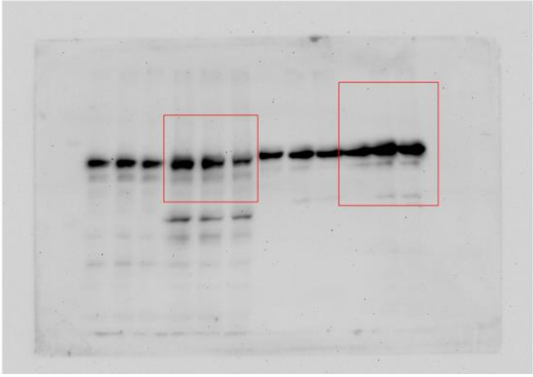

GFP-PHR-caspase8

Anti-GFP

FIG2B

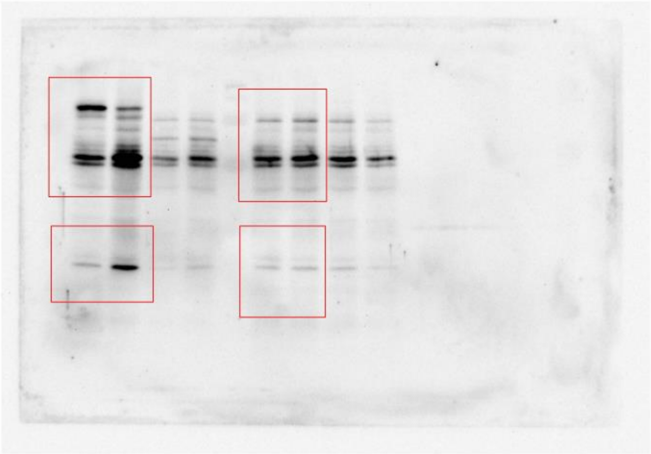

anti-caspase8+anti-p18

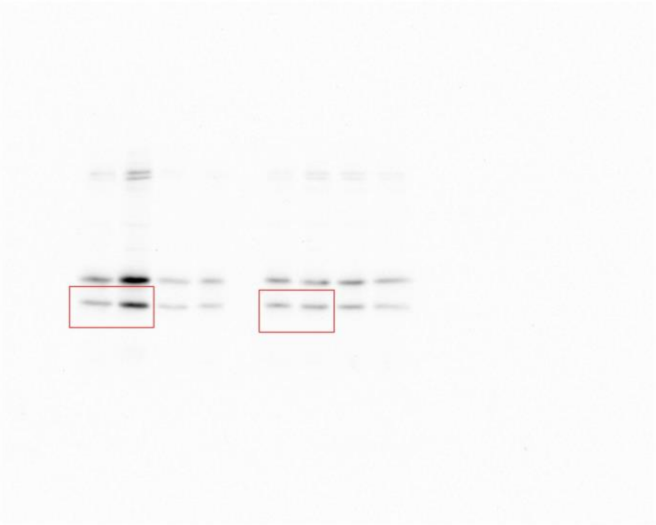

pro caspase3

active caspase3

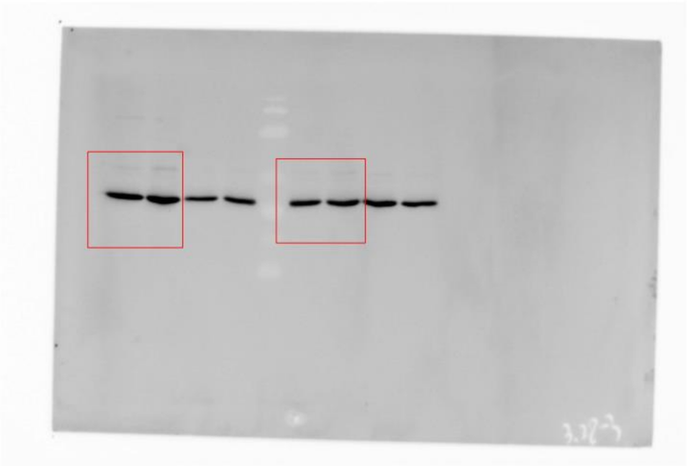

anti-actin

FIG2C

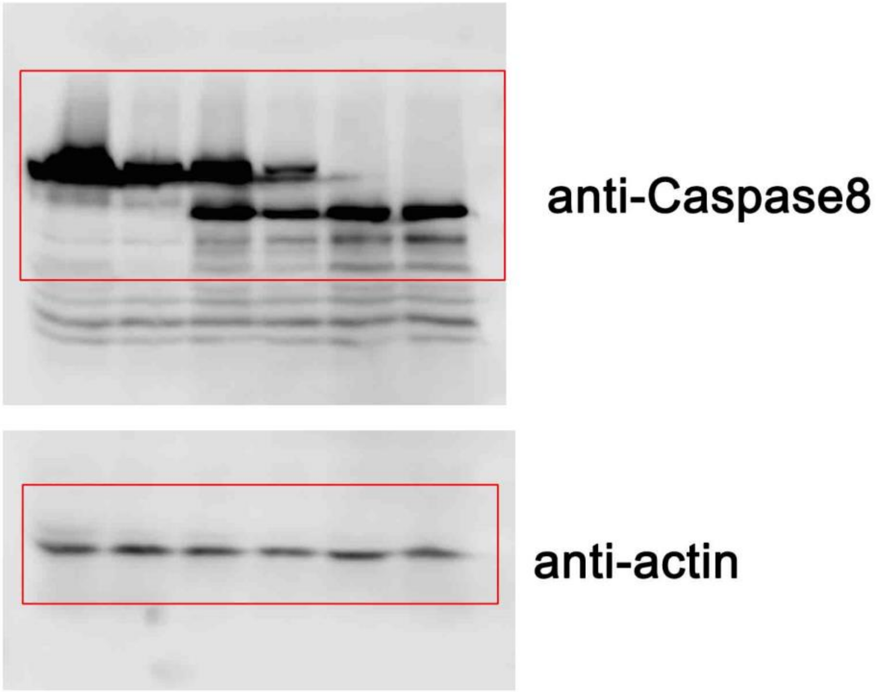

FIG2D

Table1 primers used in this study

primer name	sequence
Myc to pCI(neo) F	CTAGCCTCGAGAATTCATGGGGTTAATTAACGGTGA
Myc to pCI(neo) R	TACCACGCGTGAATTCGCTACCGTTCAAGTCTTCC
Flag to pCI(neo) F	CTAGCCTCGAGAATTCATGACTGATTACAAGGATG
Flag to pCI(neo) R	TACCACGCGTGAATTCACCTCCACCACCTCCTCCCT
GFP to pCI(neo) F	CTAGCCTCGAGAATTCATGGTGAGCAAGGGCGCCGA
GFP to pCI(neo) R	TACCACGCGTGAATTCCTTGTACAGCTCATCCATGC
CRY2PHR to pCI(neo) F	GCGTGGTACCTCTAGAATGAAGATGGACAAAAAGAC
CRY2PHR to caspase8 R	TGAAGTCCATCCCGGGTGCTGCTCCGATCATGATCT
caspase8 F	CGGAGCAGCACCCGGGATGGACTTCAGCAGAAATCT
caspase8 R	GAAGCGGCCGCCCGGGATCAGAAGGGAAGACAAGT
CIB1N to pCI(neo) F	GCGTGGTACCTCTAGAATGAATGGAGCTATAGGAGG
CIB1N R	TGAAGTCCATCCCGGGAGTGGCTTGGCCTCGTCGTG
